# Spatial distribution characteristics of tumor marker CA724 reference values in China

**DOI:** 10.1002/cam4.2176

**Published:** 2019-06-14

**Authors:** Jing Jing, Miao Ge, Ziqi Yang, Peng Li

**Affiliations:** ^1^ Institute of Healthy Geography, College of Geography and Tourism Shaanxi Normal University Xi'an Shaanxi China; ^2^ Baoji University of Arts and Sciences Baoji Shaanxi China; ^3^ Department of Epidemiology and Biostatistics, School of Public Health Xi'an Jiaotong Unversity Health Science Center Xi'an Shaanxi China

**Keywords:** CA724, gastric cancer, geographical environment factors, reference values, tumor markers

## Abstract

**Objects:**

This study aims to explore the Cancer antigen 724 (CA724) reference values spatial distribution characteristics in healthy Chinese adults. The study can provide regional reference for medical diagnosis.

**Study Design:**

The relationship between CA724 and 25 geographical environmental factors was analyzed firstly. Artificial neural network simulation training was used to construct the prediction model. The national forecast distribution map of the CA724 reference values was obtained by the geostatistical mapping method. Analyzing and exploring the influence mechanism of geographical environment factors on CA724 reference values.

**Methods:**

Collecting 34470 cases from more than 106 cities healthy adults CA724 reference values via several paper databases in 10 recent years. Correlation analysis, RBF artificial neural networks and trend surface analysis were applied to explore if there was any tendency of spatial variation. The Kriging interpolation of geostatistical analysis was developed to reveal the spatial distribution characteristics of the CA724 reference values.

**Results:**

The distribution of CA724 reference values of Chinese healthy adults shows a downward trend from south to north. CA724 reference values have negative correlations with latitude, annual sunshine duration and topsoil cation exchange capacity in clay. CA724 have positive correlations with annual mean air temperature, annual mean relative humidity, and annual precipitation amount. High temperature and high humidity environment will reduce gastrointestinal function and breeze various mold bacteria. Lack of sunshine can easily lead to vitamin C deficiency in the body. These will increase the incidence of gastrointestinal diseases and gastric cancer, then increase the CA724 value.

**Conclusion:**

CA724 reference values show spatial autocorrelation and regional variation. There are some geographical environment factors effected Chinese healthy adults CA724 reference values. Geographic factors such as sunshine, temperature, and humidity have effects on CA724 reference values can provide new ideas and directions of prevention and clinical diagnosis in the future.

## 1 INTRODUCTION

Cancer antigen 724 (CA724) is a high molecular weight mucin carcinoembryonic antigen and the molecular weight is >200 000. It has double antigenic determinant. Its level is related to tumor size, stage, and metastasis. CA724 is present in stomach, colon, pancreas, lung, and ovarian tumors for almost 85%‐95%. However, it does not exist in benign tumors, exudates, or normal human tissues.^1^ It is a broad‐spectrum tumor marker. In patients with malignant tumors of the digestive system, serum is elevated. Gastric cancer patients have higher expression.^2^ It is reported that CA724 has a sensitivity to gastrointestinal tumors of 78.8%, which is a reliable indicator for the diagnosis of gastric cancer.^3^, ^4^, ^5^ Research reported that: serum CA724 level after gastric cancer surgery is significantly lower than before surgery, it can be used for monitoring the therapeutic effect.^6^ The level of CA724 is closely related to clinical stage, tumor size, lymph node involvement and invasion and metastasis of gastric cancer, it has high specificity.^7^ The positive rate of tumor markers in gastric cancer in all stages of gastric cancer is significantly correlated with tumor stage, prognosis, and depth of tumor invasion, and may indicate distant metastasis. It can also be used as an early monitoring indicator for recurrence after radical surgery and evaluate the chemotherapy effect of gastric cancer.^8^ Obviously, the determination of the CA724 reference range is particularly important. Many factors affecting CA724 in existing research results include heredity, eating habits, lifestyle habits, sunshine, etc French researchers Martel et al^9^ have presented some known risk factors, including genetic, dietary, behavioral, and Helicobacter pylori infection in gastric cancer. A research of Korea has pointed that salt or some chemicals contained in kimchi and soybean pastes, which are increased risk of gastric cancer.^10^ You et al^11^ concluded some influencing factors of gastric cancer such as H. pylori infection, cigarette smoking, and low levels of dietary vitamin C. In Japan, the negative correlation coefficients of male age adjusted stomach cancer mortality rate with yearly sunshine duration was significant, but no correlation was found with yearly average temperature. Calcium has antiphlogistic effect, because of its capillary permeability reducing action, which may also prevent lodging of tumor cells in hematogenous metastasis of cancer in microcirculation system. The sunshine duration may be concerned with calcium absorption through its action of vitamin D production on skin.^12^ An investigation in Jiangsu province of China, risk factors for gastric cancer include family history of cancer, fast eating, eating salty food, hot food, male smoking, etc^13^ The CA724 shows higher value in patients with gastric cancer.

The human and geographical environment, however, is an organic and interactive whole. A lack of studies exists for the relationship between CA724 reference values and the geographical environment. This study focuses on exploring the relationship between CA724 reference values and geographical environmental factors, revealing the spatial characteristics of CA724 reference values in China. It can provide scientific basis for clinical diagnosis and treatment. This study provides a regional basis for clinical diagnosis and treatment.

## 2 MATERIALS AND METHODS

### 2.1 Study area and subjects

The 34 470 cases of Chinese healthy adults with CA724 reference values were collected. Organizing CA724 reference values from more than 190 administrative units in 106 cities and regions via several paper databases (2008‐2018) (http://www.cnki.net). The cities are selected randomly. Selecting the units according to the unified sampling method. Western cities have less data than the eastern ones. There are 115 units from eastern regions, 42 units from middle regions, and 33 units from western regions due to disparities of population density. However, Hong Kong Special Administrative Region, Macao Special Administrative Region and Taiwan province were not involved in this study. The ratio of males (18075) to females (16375) was 1:1.1. The subjects were all apparently healthy with the inclusion criteria being normal weight and height, that is, (body mass index [BMI] between 18.5 and 25.0 kg/m^2^), normal blood pressure and normal blood glucose after 8 hours' fasting. In addition, *t* test was employed to test the disparities of CA724 reference values among different regions. *T* test result is *P* ¼ 0.001 < 0.05, which indicate there is a significant difference of CA724 reference values among different regions. Serum samples were selected from healthy individuals in each unit during the same period to exclude gastric cancer or other diseases that could cause changes in CA724.

### 2.2 Geographical Environment variables

Information on 4 classes of geographical environment factors were collected concluding spatial indicators, topographical indicator, meteorological indicators, and soil indicators. Spatial indicators contain Longitude (*X*_1_) and Latitude (*X*_2_). Topographical indicator contains Altitude (*X*_3_). Spatial location and terrain indices from relevant maps and dictionaries. Meteorological indicators contain 6 indexes (*X*_4_‐*X*_9_). Meteorological index provided by the meteorological data sharing service system in China (http://www.cma.gov.cn). Soil indicators contain 14 indexes (*X*_10_‐*X*_25_). Soil indexes obtained from the Harmonious World Soil Database (HWSD) of Vienna International Institute of Applied Systems (IIASA) supplied by the Food and Agriculture Organization (FAO) (http://www.fao.org/nr/land/soils/harmonized-world-soil-database/zh/). In all, 25 different geographic locations and environmental variables (referred to by abbreviations *X*_1_‐*X*_25_) were used as shown in Table 1.

**Table 1:** Geographical environment factors

*X_n_*	Geographical environment factors	*X_n_*	Geographical environment factors	*X_n_*	Geographical environment factors
*X*_1_	Longitude (°)	*X*_10_	Topsoil sand percentage (% wt.)	*X*_19_	topsoil cation exchange capacityin silt (cmol/kg)
*X*_2_	Latitude (°)	*X*_11_	Topsoil powder percentage (% wt.)	*X*_20_	Topsoil base saturation (%)
*X*_3_	Altitude (m)	*X*_12_	The percentage by overburden clay (% wt.)	*X*_21_	Topsoil exchangeable base (cmol/kg)
*X*_4_	Annual sunshine duration (h)	*X*_13_	Topsoil reference capacity (kg/dm^3^)	*X*_22_	Topsoil carbonate (lime) content (cmol/kg)
*X*_5_	Annual mean temperature (℃)	*X*_14_	Overburden soil bulk density (kg/dm^3^)	*X*_23_	Topsoil sulfate (gypsum) content (cmol/kg)
*X*_6_	Annual mean relative humidity (%)	*X*_15_	Topsoil fertile volume percent (% vol.)	*X*_24_	Surface soil alkalinity (cmol/kg)
*X*_7_	Annual precipitation amount (mm)	*X*_16_	Topsoil organic carbon (% wt.)	*X*_25_	topsoil salinity (dS/m)
*X*_8_	Annual range of temperature (℃)	*X*_17_	Topsoil PH		
*X*_9_	Annual mean wind speed (m/s)	*X*_18_	Topsoil cation exchange capacity in clay (cmol/kg)		

## 3 METHODS

### 3.1 Correlation analysis

According to the principle and method of correlation analysis, the correlation between CA724 reference values and 25 geographical factors were analyzed. The principle analysis can find out the relevant geographical factors. The study was conducted using SPSS 22.0 software by comparing CA724 reference values and geographical environment factors. The correlation was considered statistically significant when the *P*‐value was at the 95% confidence interval, and it was marked as *. The correlation was considered as strongly statistically significan when the *P*‐value achieved a 99% confidence interval *t*, and it was marked as **.

### 3.2 Artificial neural networks

Artificial neural network is based on the structure of the brain tissue. The mechanism of understanding based on the structure and behavior of an engineering system.^14^, ^15^ In the prediction function by the scientific community recognized as superior to the traditional statistical prediction method. It has a better application of expression in medical clinical diagnosis, medical imaging, prognosis, and clinical decision‐making analysis and so on.

This study uses radial basis function neural network (RBFANN) for modeling. The basic idea of RBFANN is to use RBF as a function of hidden nodes. When the input layer directly passes signals to the hidden layer, the input vector can be directly mapped to the hidden space without connecting through the weights. Thus making the hidden layer node output.^16^ *n*_i_ is represented the number of input layer nodes of the 3‐layer forward neural network. *n*_h_ is expressed the number of hidden layer nodes. *n*_o_ is represented the number of output layer nodes. In order to find a more suitable number of hidden layer nodes in a large number of experiments and practical applications, the range of ideal hidden layer nodes appears to be:s

(1) $$\frac{n_{i}+n_{o}}{2}\leq n_{h}\leq \left(n_{i}+n_{o}\right)+10$$

After confirming the range of the number of hidden layer nodes, the RBFANN corresponding to the number of hidden layer nodes in the range is trained. After that training the same sample and comparing the test results.

Whether the distribution of CA724 reference values in this study are linear or nonlinear, relying on statistical methods or human experience to determine the results are not necessarily realistic. Therefore, the neural network is used to compare the linear model with the nonlinear model in order to predict the nonlinear relationship between the CA724 reference values and the geographical environment. RBFANN is used to build network models. The reference values of CA724 are predicted by the geographical environment factors which have correlations with CA724.

### 3.3 Spatial statistics analysis

Spatial statistical analysis reveals spatial changes and features through the distribution of data sets characterized by geographic attributes. The core concept is derived from Tobler's first law of geography. Geographic phenomena or objects exhibit spatial dependence and variation characteristics.^17^ Under the long‐term influence of the geographical environment, the adaptation of bodily functions has led to various regional characteristics.

### 3.4 Spatial autocorrelation

Spatial autocorrelation is mainly used to analyze the correlation and dependence between geographical phenomena or attributes and their adjacent units in a certain place. Its purpose is to reveal the dependence.^18^, ^19^ Moran's *I* is the main indicators for assessing spatial dependence and correlation.^20^, ^21^ We used Moran's *I* index to assess the degree of dependence and correlation of CA724 reference values in space. The threshold range of Moran's *I* is [−1, 1]. When Moran's *I* index is greater than zero, it represents a positive statistical correlation between spatial objects. Conversely, when it is less than zero, there is a negative correlation. When the index is the same as zero, the mean shows the balenced distribution of the spatial objects.^22^, ^23^

The significance of the Moran's *I* index is tested by the standard statistic‐*Z* value. We suggest that the determination of significance depends on testing the *Z* value with the *P* value. It is statistically significant if the *Z* value is greater than zero. However, it indicates a negative statistical significance if the *Z* value is less than zero. When the *Z* value is equal to zero, it presents a random distribution.

### 3.5 Trend surface analysis

Trend surface analysis is a multivariate analysis of data fitted by abstract mathematical curves based on least squares.^24^ In general, this surface includes trend surface and residual surface. The trend surface mainly reflects the macroscopic distribution of geographic datasets, while the residual surface mainly reflects the influence of random factors on the overall distribution of microscopic space. Therefore, it is necessary to reduce the residual value as soon as possible to eliminate the influence of random variables. The purpose of the random variable reflects the spatial distribution of the sample and improves the simulation accuracy.^25^ Using trend surface analysis to analyze the global trend of CA724 reference prediction data and establish a three‐dimensional view. Each point in the data set is projected on an orthogonal plane in the east‐west and north‐south directions. Then based on the corresponding projections, the best fit lines are determined and the corresponding presence trends are described by it.

### 3.6 Spatial distribution

In order to reveal the distribution law of Chinese adults' CA724 reference values, 2322 cities in China were selected as the observation points. The predicted values of CA724 were calculated. The Kriging interpolation was performed by using the spatial analysis module of ArcGIS software and the spatial distribution map was output.^26^, ^27^ Kriging interpolation method considers the shape information of the sample point, the spatial orientation, and the structural information provided by the variogram. Then estimating the prediction data unbiased based on the sample data of CA724. Kriging interpolation can be used to obtain the spatial distribution trend of the CA724 reference values in China.

## 4 RESULTS

### 4.1 Correlation analysis

The correlations between CA724 and geographical environment factors are shown in Table 2. It shows that the CA724 is significantly correlated with geographical environment factors in China. Apparently, the correlation between CA724 and *X*_2_, *X*_5_, *X*_7_, *X*_18_ was significant (*P* = 0.015, 0.037, 0.048, 0.040, <0.05); the correlation between CA724 and *X*_4_, *X*_6_ was very significant (*P* = 0.003, 0.008, <0.01). The CA724 has a negative correlation with Latitude (*X*_2_), Annual sunshine duration (*X*_4_), and topsoil cation exchange capacity in clay (*X*_18_), but has a positive correlation with annual mean air temperature (*X*_5_), annual mean relative humidity (*X*_6_) and annual precipitation amount (*X*_7_).

**Table 2:** Correlation and significance of CA724 in healthy Chinese adults

Geographical factors (*X_n_*)	*t*	*P*
*X*_1_	−0.025	0.733
*X*_2_	−0.176	0.015*
*X*_3_	0.028	0.699
*X*_4_	−0.217	0.003**
*X*_5_	0.151	0.037*
*X*_6_	0.193	0.008**
*X*_7_	0.144	0.048*
*X*_8_	−0.124	0.088
*X*_9_	−0.049	0.499
*X*_10_	−0.076	0.298
*X*_11_	0.106	0.145
*X*_12_	−0.024	0.738
*X*_13_	−0.024	0.744
*X*_14_	−0.016	0.822
*X*_15_	0.117	0.108
*X*_16_	0.026	0.724
*X*_17_	0.074	0.310
*X*_18_	−0.149	0.040*
*X*_19_	−0.100	0.172
*X*_20_	0.062	0.398
*X*_21_	0.078	0.286
*X*_22_	0.066	0.363
*X*_23_	−0.100	0.169
*X*_24_	−0.049	0.500
*X*_25_	0.095	0.151

* Correlation is significant at the 0.05 level (2‐tailed).

** Correlation is significant at the 0.01 level (2‐tailed).

### 4.2 Artificial neural networks

According to formula (1), the number of geographic factors associated with the CA724 reference values is 6, the corresponding input layer node number is *n*_i_ = 6, and the number of output layer nodes is *n*_o_ = 1, so the corresponding hidden layer node number *n*_h_ is in the range of [4, 17].

Importing the training data into SPSS Clementine 12.0 and establish a data stream. Connecting the neural net node in the modeling module to the appropriate location of the data stream. Establishing the RBFANN model for each node number to repeatedly train the same sample data. The test samples are predicted by each model. The analysis node is added to the prediction result node to analyze the prediction results. (Table 3) Calculating the Mean Absolute Error (MAE) and Mean Squared Error (MSE) of the RBFANN model corresponding to the number of hidden nodes. According to the results, when the number of hidden layer nodes is 5, the prediction effect is best. Therefore, the model corresponding to the number of five hidden layer nodes is selected as the RBFANN prediction model of the CA724 reference values.

**Table 3:** Mean Absolute Error (MAE) and Mean Squared Error (MSE) of radial basis function neural networks with different node of hidden layer

Node of hidden layer	MAE	MSE
4	1.075	1.411
5	1.046	1.379
6	1.052	1.396
7	1.087	1.405
8	1.071	1.392
9	1.068	1.388
10	1.073	1.388
11	1.044	1.389
12	1.101	1.424
13	1.076	1.438
14	1.110	1.430
15	1.123	1.468
16	1.066	1.412
17	1.088	1.454

### 4.3 Spatial autocorrelation

**Figure 1:**
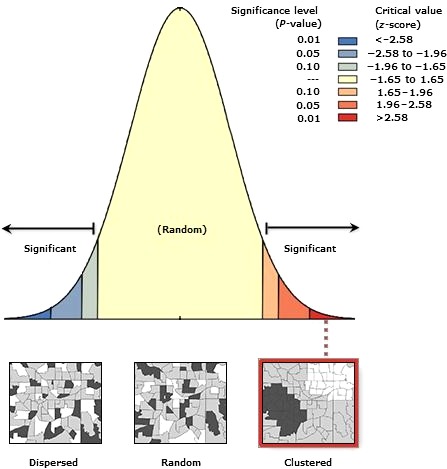
The spatial distribution of CA724 is clustered or dispersed

As shown in Figure 1 and Table 4, Moran's *I* was equal to 2.301783; the *Z*‐value was 20.934111 by the test of auto‐correlation. The result was greater than 2.58 in the 99% confidence interval. The *P*‐value <0.01. In addition, Figure 1 showed a significant autocorrelation of the CA724 reference values in spatial distribution. It is indicated that the spatial disparities generated the regional distribution of the reference values of CA724. CA724 reference values were influenced by spatial disparities and geographical factors.

**Table 4:** Spatial autocorrelation report—Moran's results

Moran *I*	Expected	Index variance	*Z*‐value	*P*‐value
2.301783	−0.007299	0.012167	20.934111	0.000000

### 4.4 Trend surface analysis

A trend surface analysis was developed to explore the spatial trend of the CA724 reference values. We can clearly see 2 curves reflecting the reference values of CA724 in a 3‐dimensional grid. The red curve was designated as the direction of from west to east, and the blue curve was designated as the direction from south to north. The CA724 reference values are gradually reduced from south to north, which was consistent with the spatial distribution. Speculatively, there is a distinctly regional distribution in space.

**Figure 2:**
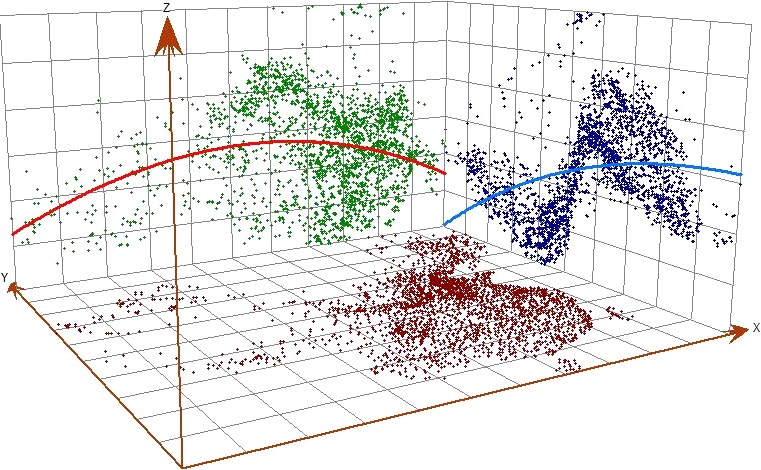
CA724 in healthy Chinese adults trend analysis chart. Red line is from West to East, Blue line is from North to South.

As presented in Figure 2, the CA724 values for healthy adults along the longitude it first increased from west to east and then decreased slightly in the extreme (the red curve), while it showed a decreasing trend from south to north along the latitude (the blue curve). With the ordinate (*x* axis) depicting longitude, the abscissa (*y* direction) latitude and *z* the CA724 concentration, it can be seen that the CA724 reference values first increase in the western direction along the longitude and then decrease in the eastern direction at the end (the red slope), while they decrease in the northern direction along the latitude (the blue curve).

### 4.5 Spatial distribution

**Figure 3:**
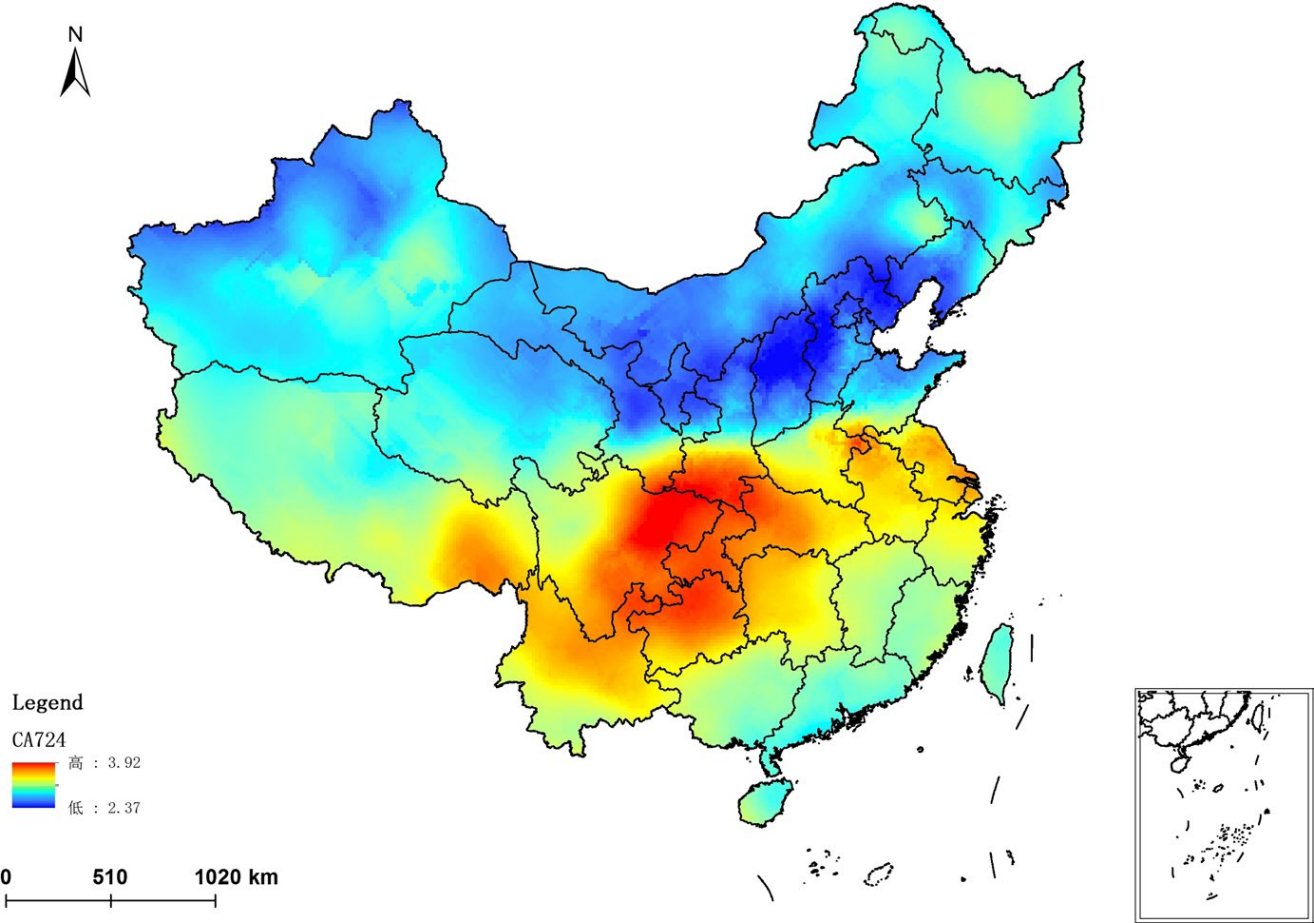
Spatial tendency chart of the reference values for CA724 of Chinese healthy adults

In order to clearly and accurately show the geographical distribution law of the CA724 of Chinese healthy adults. Using geostatistics module in ArcGIS software, 2322 Chinese cities were located in Chinese map. The Kriging interpolation was conducted after analyzing, screening, and transforming the data which were predicted by the optimal predictive model. The spatial tendency chart of the Chinese healthy adults' CA724 reference values was output (Figure 3).

It can be seen from Figure 3, the distribution of Chinese healthy adults' CA724 reference values shows a downward trend from south to north. CA724 reference values have negative correlations with latitude (*X*_2_) annual sunshine duration (*X*_4_) and topsoil cation exchange capacity in clay (*X*_18_). They have positive correlations with annual mean air temperature (*X*_5_), annual mean relative humidity (*X*_6_), and annual precipitation amount (*X*_7_). The trend consistent with the distribution law of annual mean relative humidity and in contrary to annual sunshine duration distribution of China. With the increase of latitude and annual sunshine duration, the CA724 reference values of Chinese healthy adults decreased. With the increase of annual mean air temperature (*X*_5_), annual mean relative humidity (*X*_6_) and annual precipitation amount, the CA724reference values increased in Chinese healthy adults. This shows that Chinese healthy adults CA724 reference values and geographical environment factors have a correlation in spatial distribution.

## 5 DISCUSSION

From the results of this study we can see that geographical environment factors relatively relevant with the Chinese healthy adults' CA724 reference values including latitude, annual sunshine duration, annual mean air temperature, annual mean relative humidity, annual precipitation amount, and topsoil cation exchange capacity in clay. The CA724 reference values have negative correlations with latitude, annual sunshine duration, and topsoil cation exchange capacity in clay, but have positive correlations with annual mean air temperature, annual mean relative humidity and annual precipitation amount.

### 5.1 The influence of temperature for CA724

In epidemiology, the incidence of digestive ulcers increases from north to south. This is consistent with the findings of this paper. Researchers of Japan have acquired data for infectious gastroenteritis cases and weather variability in Fukuoka, from 1999 to 2007 and used time‐series analysis to assess the effects of weather variability on infectious gastroenteritis cases, adjusting for confounding factors. In total, 422 176 Infectious gastroenteritis cases were reported during the 9‐year study period. The weekly number of infectious gastroenteritis cases increased by 7.7% (95% CI 4.6‐10.8) for every 1°C increase in the average temperature.^28^ A few studies in high‐income countries have investigated the relationship between ambient temperature or precipitation and the occurrence of gastroenteritis. In most of the cases, hot temperatures and heavy precipitation events have been related to increases in infections. The findings suggest an important role of ambient temperatures, especially hot temperatures, in increasing gastroenteritis hospitalizations.^29^ Various studies have shown that the incidence of gastrointestinal diseases increases with increasing temperature. High temperature conditions will reduce the secretion of digestive juices such as saliva, gastric juice, pancreatic juice, bile, and intestinal fluid. The digestive juices contain substances necessary for the digestion process of the food, such as free hydrochloric acid, protease, lipase, amylase, etc These substances can reduce the digestive function of the stomach. In high temperature environment, the increase of sweating causes a large loss of sodium chloride, and because the chloride ion of free hydrochloric acid in gastric juice comes from the blood, the acidity of gastric juice is also significantly reduced.^30^ High temperature environment can also cause the gastric emptying to accelerate, so that the chemical digestion process of food can not be fully carried out. Because of the effects of temperature on the central nervous system of people, high temperatures often lead to loss of appetite and irregular diet.^31^ People eating melon and fruit cold drinks in high temperature weather increases the risk of gastrointestinal diseases. Affected by many factors above, high temperature can lead to high incidence of gastrointestinal diseases, resulting in an increase in CA724.

### 5.2 The influence of humidity for CA724

The relative humidity of about 50% is the most unfavorable humidity condition for the disease of the digestive system. The threshold range corresponds to the most comfortable humidity range that the human body feels.^32^ The annual average relative humidity in southern China just fits this range. Increased humidity causes food to become mildewed, and the growth of various molds greatly increases the incidence of gastric cancer. High temperature and high humidity are easy to breed various bacteria. Fungi such as Aspergillus flavus, Bacillus cereus, Botox, Salmonella typhimurium, etc are beneficial for growth and reproduction of digestive tract diseases. The fact that Aspergillus flavus is found in soya bean cakes raises the possibility that aflatoxins produced by this mold which grows on the soya bean cakes (from which soya bean paste is made) may be a possible etiologic factor in the high incidence of stomach cancer.^33^ Infection of Salmonella typhimurium (Salmonella typhi) can lead to various organ diseases. This research first proposed that Salmonella typhi‐infection could result in gastric oxidative stress and hemorrhagic ulcers.^34^ Bacterial growth and reproduction are inseparable from high temperature and high humidity weather conditions, which can enable bacteria to grow and multiply better. In short, as the temperature and humidity increase, the proliferation of various molds increases, leading to an increase in the incidence of various gastrointestinal diseases, resulting in an increase in CA724. Therefore, the CA724 reference values of Chinese healthy adults are higher in the south than in the north.

### 5.3 The influence of sunshine for CA724

Studies have shown that human body under the action of sun through the skin synthesis of large amounts of vitamin D. More than 80% of vitamin D from the sun in the ultraviolet radiation less through the source of nutrients provided, because vitamin D from the natural food are very few except fish. The factors that affect the skin synthesis of vitamin D are mainly due to adequate exposure to sunlight and dietary supplementation to achieve adequate levels of vitamin D.^35^, ^36^ Vitamin D not only maintains calcium and bone homeostasis, but also mostly inhibits tumor genesis, invasion, and metastasis through activation of vitamin D receptor. accumulating evidence from gastric cancer cells, animal models, and clinical trials suggest that vitamin D deficiency may increase the risk and mortality of gastric cancer.^37^ Vitamin D‐3 upregulated protein 1 (VDUP1) is a potent tumor suppressor whose expression is dramatically reduced in various types of human cancers, including gastric cancer.^38^ Ample sunshine can provide the body with adequate vitamin D, which can inhibit the risk of stomach cancer. In contrast, fewer sunshine hours in the year will increase the incidence of Helicobacter pylori and gastric cancer, resulting in a high CA724 value.

### 5.4 The influence of soil for CA724

Generally, the effect of soil on the human body is relatively indirect. It is usually caused by the influence of the soil on the crop, then the re‐effect of the crop or food on the person. The soil cation exchange amount directly reflects the soil fertilizer retention, fertilizer supply performance and buffer capacity. In the case that the normal soil is not subjected to severe heavy metals or other pollution. The stronger soil topsoil clay cation exchange energy is the better soil fertility is. This will help plants absorb, purify water, and grow their crops healthily. A healthy crop or food will be beneficial to the health of the human gastrointestinal tract. So healthy conditions of people will show a low CA724 value.

## 6 CONCLUSIONS

The present research provides a new view and insight in medical geography science through studying and exploring. The geographical environment and the human body are an organic integration. Various environments cause differences in our bodies, but CA724 reference values show similar and spatial dependence. We have probed the relationship between CA724 reference values of Chinese healthy adults and geographical environmental factors. In this paper, we choose the spatial regions of China as the research object, using the correlation analysis, spatial autocorrelation analysis, RBF Artificial neural networks, trend surface analysis, spatial analysis, and other methods to predict models. This study also explored the spatial distribution characteristics of CA724 reference values for clinical practice and future study. The laws of the distribution of Chinese healthy adults CA724 reference values shows the overall trend that a downward trend from south to north. There are some geographical environment factors effected Chinese healthy adults CA724 reference values. The spatial distribution of Chinese healthy adults CA724 reference values is consistent with latitude, annual sunshine duration, annual mean air temperature, annual mean relative humidity, annual precipitation amount, and topsoil cation exchange capacity in clay distribution.

Geographical environmental factors encompass a wide range of content. Many factors such as the ecological environment in which humans live, the water quality conditions of domestic water, the air quality in the living environment, the soil conditions, and many geographical conditions affect the changes in the reference values of medical indicators. The geographical environmental factors selected in this paper are still not comprehensive. In the future research, we will also consider increasing geographical environment factors such as water environment, atmospheric environment, and human environment which have impacts on human health. It is currently known that changes in the geographical environment do have an impact on human health and physiological indicators. How the geographical environment factors affect and act on the human body remains to be further scientifically and deeply explained. In the future, we will further try to analyze or simulate the mechanism of influence mechanism by strengthen the professional study of medicine and geography knowledge. On the one hand the study can provide scientific reference for medical diagnosis, on the other hand it can enrich the contents of medical geography. In future studies, it is expected that the study will be further refined, in order to provide new ideas and scientific basis for the prevention, early detection and treatment of cancer such as gastric cancer.

## CONFLICT OF INTEREST

None of the authors have any financial or other potential conflict of interest for this study.

## ETHICAL APPROVAL

The study was approved by Medical Ethics Committee of Second Affiliated Hospital of Xi'an Jiaotong University medical school (2010‐LS‐009).
